# Rh-Catalyzed reductive Mannich-type reaction and its application towards the synthesis of (±)-ezetimibe

**DOI:** 10.3762/bjoc.12.157

**Published:** 2016-07-27

**Authors:** Motoyuki Isoda, Kazuyuki Sato, Yurika Kunugi, Satsuki Tokonishi, Atsushi Tarui, Masaaki Omote, Hideki Minami, Akira Ando

**Affiliations:** 1 Faculty of Pharmaceutical Sciences, Setsunan University 45-1, Nagaotoge-cho, Hirakata, Osaka 573-0101, Japan; 2 Faculty of Pharmaceutical Sciences, Hiroshima International University, 5-1-1 Hirokoshingai, Kure, Hiroshima 737-0112, Japan

**Keywords:** β-lactam, ezetimibe, reductive Mannich-type reaction, rhodium–hydride, zinc enolate

## Abstract

An effective synthesis for *syn*-β-lactams was achieved using a Rh-catalyzed reductive Mannich-type reaction. A rhodium–hydride complex (Rh–H) derived from diethylzinc (Et_2_Zn) and a Rh catalyst was used for the 1,4-reduction of an α,β-unsaturated ester to give a Reformatsky-type reagent, which in turn, reacted with an imine to give the *syn*-β-lactam. Additionally, the reaction was applied to the synthesis of (±)-ezetimibe, a potent β-lactamic cholesterol absorption inhibitor.

## Introduction

The Mannich reaction is an important and classical C–C bond-forming reaction between an enolizable carbonyl compound and an imine to give the corresponding β-aminocarbonyl compound. For example, Shibasaki and his colleague reported the asymmetric Mannich reaction using a Lewis acid catalyst [1]. (L)-Proline is known as an excellent promoter for the Mannich reaction [2–6], and besides this, the reaction of the silyl enol ether derivatives with imines was used as an effective method [7–9]. In this situation, a wide variety of Mannich-type reactions have been reported to give β-amino esters and/or β-lactams by using metal enolates [10–17].

**Scheme 1:**
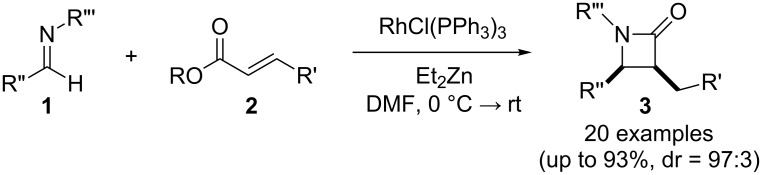
The synthesis of *syn*-β-lactams using a reductive Mannich-type reaction.

**Scheme 2:**
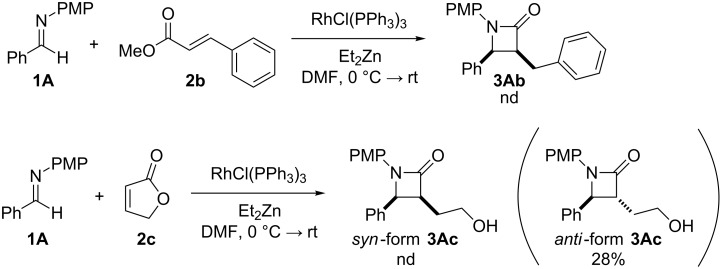
Previous results using β-substituted α,β-unsaturated esters.

In contrast, most of reductive Mannich-type reactions using imines and α,β-unsaturated carbonyl compounds gave β-amino esters, but there are only a few reports for a direct synthesis of β-lactams by reductive Mannich-type reactions [14,18–21]. We recently reported a reductive Mannich-type reaction using a combination of RhCl(PPh_3_)_3_ and Et_2_Zn to give the corresponding *syn*-β-lactams from α,β-unsaturated esters and imines in good to excellent yields together with a small amount of the β-amino esters (Scheme 1) [22–23]. The reaction is very noteworthy because the formation of *syn*-β-lactams is particularly rare [24–27]. Additionally, various imines can be used for this reaction, and the corresponding β-lactams showed very high *syn* selectivity. However, some of the reactions using β-substituted α,β-unsaturated esters did not afford the products or gave the products in very low yield (Scheme 2). Herein, we report an expansion for our previous reductive Mannich-type reaction and mechanistic studies for the stereoselectivity of this reaction. Finally the method is applied for the new synthesis of ezetimibe, a potent β-lactamic cholesterol absorption inhibitor.

## Results and Discussion

First of all, we tried to solve the drawbacks of the previously reported method and found that changing the catalyst to [RhCl(cod)]_2_ greatly improved the reaction. The results from the reaction of the [RhCl(cod)]_2_ catalyst with various α,β-unsaturated esters are summarized in Table 1. Most of the synthesized β-lactams were obtained with dramatically improved yields, although the β,β-disubstituted α,β-unsaturated ester did not give the product owing to the bulkiness of the β-position (Table 1, entries 1–8). It is interesting that ethyl sorbate (**2g**) gave the corresponding *anti*-β-lactam **3Ag** in a moderate yield but with an *anti*-β-lactam stereochemistry indicating that the reaction proceeded via 1,6-reduction, then the resulting nucleophile was trapped by the imine at the α-position (Table 1, entry 9). By using [RhCl(cod)]_2_, the α,β-unsaturated lactone was also converted to the product with a small improvement in the yield (Table 1, entry 11). Acrylamide **2i** also gave the corresponding β-aminoamide **4Ai** in a low yield (Table 1, entry 12).

**Table 1:** Rh-catalyzed Mannich-type reaction using various α,β-unsaturated esters.

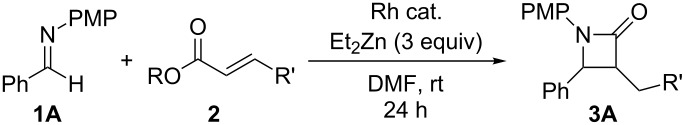				

Entry	Substrate **2**	Rh cat. (mol %)	Product	Yield (%)^a^

1	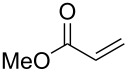 **2a**	RhCl(PPh_3_)_3_ (2)	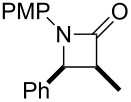 **3Aa**	88 [*syn*/*anti* = 96:4]^b^
2		[RhCl(cod)]_2_ (1)		78 [*syn*/*anti* = 88:12]^b^
3	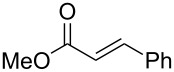 **2b**	RhCl(PPh_3_)_3_ (4)	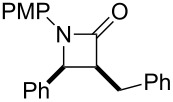 **3Ab**	nd
4		[RhCl(cod)]_2_ (2)		66^c^
5	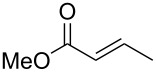 **2d**	RhCl(PPh_3_)_3_ (2)	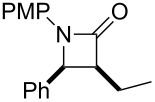 **3Ad**	34^c^
6		[RhCl(cod)]_2_ (2)		77^c^
7	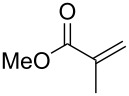 **2e**	[RhCl(cod)]_2_ (2)	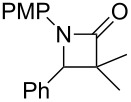 **3Ae**	98
8	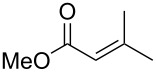 **2f**	[RhCl(cod)]_2_ (2)	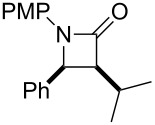 **3Af**	nd
9	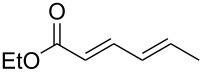 **2g**	[RhCl(cod)]_2_ (2)	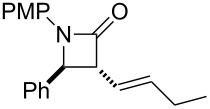 **3Ag**	59^d^ [*E*/*Z* = 93:7]^e^
10	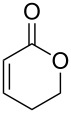 **2h**	RhCl(PPh_3_)_3_ (2)	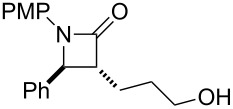 **3Ah**	11^d^
11		[RhCl(cod)]_2_ (2)		20^d^
12	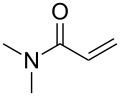 **2i**	[RhCl(cod)]_2_ (2)	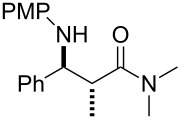 **4Ai**	27 [*syn*/*anti* = 18:82]^b^

^a^Isolated yield. ^b^Diastereomeric ratio [*syn*/*anti*] after purification. ^c^The *syn* product was obtained as the sole product. ^d^The *anti* product was obtained as the sole product. ^e^*E*/*Z* ratio by ^1^H NMR.

In the reaction using α,β-unsaturated lactone **2h**, the β-lactam *anti*-**3Ah** that has a hydroxy group on the side chain was obtained in a low yield (Table 1, entry 11). This result was of interest because the reaction is applicable to the synthesis of ezetimibe. Ezetimibe is an inhibitor of the cholesterol transporter Niemann–Pick C1 Like 1 Protein (NPC1L1), and is used as a strong cholesterol absorption inhibitor that reduces plasma low-density lipoprotein fraction (LDL-C) [28–30]. Recent studies on the function of NPC1L1 suggest that NPC1L1 may be involved with the internal uptake of the hepatitis C virus (HCV) and absorption of vitamin K [31–32]. Although it has a simple mono β-lactamic structure and appears easy to synthesize, there are relatively few reports for the synthesis of ezetimibe because of its three asymmetric centers [33–35].

First, we examined the reaction conditions using a pilot reaction using imine **1B** and 5,6-dihydro-2*H*-pyran-2-one (**2h**) (Table 2). Unfortunately, the reaction conditions as above gave a complex mixture that did not contain the desired products as shown in Table 2, entry 1. Heating the reaction mixture at 80 °C gave β-aminolactone **4Bh** in a very low yield (Table 2, entry 2). When DME and THF were used as the solvents, the desired β-lactam was obtained in low yields (Table 2, entries 3 and 4, respectively). We hypothesized that the low yield was caused by the instability of **2h** and the low reactivity of imine **1B** under the reaction conditions. Therefore, we added a Lewis acid to assist in the formation of the iminium salt in an effort to improve the reactivity. It was clear that Et_2_Zn was only working as a hydride source but was ineffective as the Lewis acid. Instead when zinc chloride (ZnCl_2_) was added into the mixture, the yield was improved to 30% (Table 2, entry 5). BF_3_·Et_2_O was found to be the most suitable Lewis acid, and the desired β-lactam (*anti*-**3Bh**) was obtained in moderate yield (Table 2, entry 9).

**Table 2:** Examination of the reaction conditions using a pilot reaction.

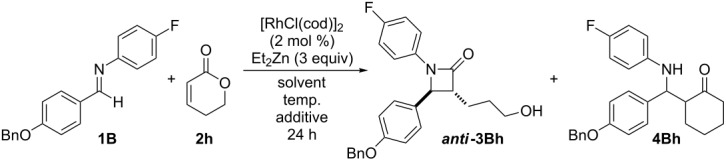					

Entry	Solvent	Temp.	Additive (equiv)	Yield (%)^a^	

				**3Bh**	**4Bh**

1	DMF	0 °C → rt	none	0	0
2	DMF	80 °C	none	0	8
3	DME	0 °C → rt	none	17	0
4	THF	0 °C → rt	none	20	0
5	THF	rt	ZnCl_2_ (1.2)	30	0
6	THF	rt	ZnCl_2_ (10 mol %)	19	0
7	THF	rt	InBr_3_ (1.2)	0	0
8	THF	rt	Al(OiPr)_3_ (1.2)	0	9
9	THF	rt	BF_3_·Et_2_O (1.2)	46	0

^a^Isolated yield.

**Scheme 3:**
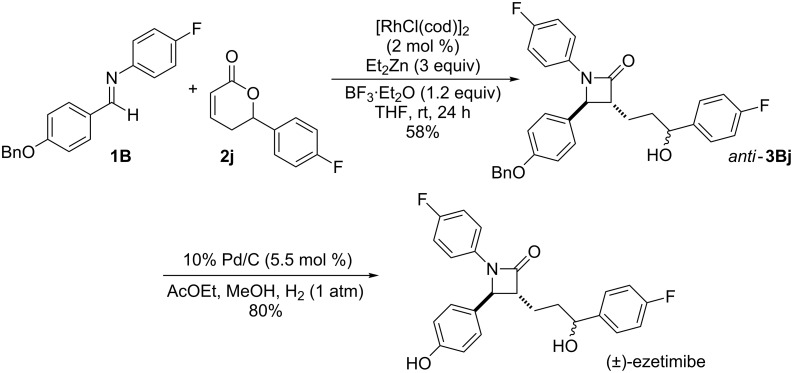
A new synthetic route for ezetimibe.

Based on these results, we applied this reaction to the synthesis of ezetimibe (Scheme 3). The reaction of imine **1B** with lactone **2j** proceeded smoothly and gave the desired β-lactam product, *anti*-**3Bj**, in 58% yield. Fortunately, since **2j** was stable compared to **2h** under the reaction conditions, the yield of β-lactam *anti*-**3Bj** was somewhat improved. After deprotection using Pd/C and H_2_ the target product, (±)-ezetimibe was obtained in 80% yield and its overall yield was 46%.

**Figure 1:**
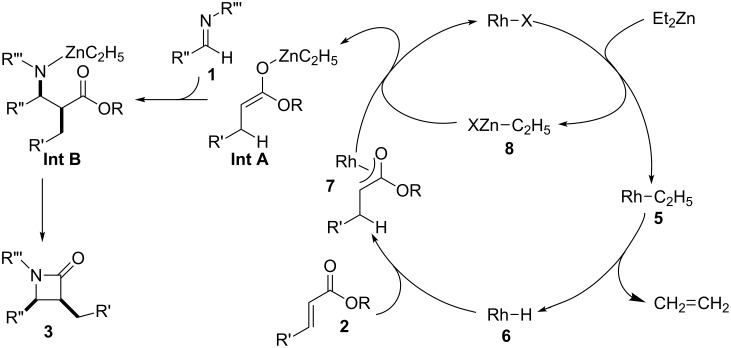
Plausible mechanism for the Rh-catalyzed reductive Mannich-type reaction.

In some of our previous publications [22–23,36], we proposed a reaction mechanism as shown in Figure 1. In the initial step, the Rh catalyst reacted with Et_2_Zn to give a rhodium–hydride complex **6** via the elimination of ethylene from the rhodium–ethyl complex **5**. The formation of rhodium enolate **7** involved the 1,4-reduction of α,β-unsaturated ester **2** by **6** and the transmetalation with a zinc species to give the Reformatsky-type reagent **Int A**. This intermediate **Int A** reacts immediately with the imine to give the corresponding intermediate **Int B**. Subsequently, intramolecular cyclization of **Int B** gave the desired β-lactam **3**. On the other hand, the possibility of alternative mechanism could not be denied which concerned with the formation of ketene from **Int A** to give the β-lactam, directly. However, we thought that the reaction proceeded through the above mechanism, because our previous results using similar conditions did not give the corresponding products such as β-propiolactones. It is well known that the substituents on the α- or β-position of α,β-unsaturated carbonyl compounds affect the yields and stereoselectivities [37]. A rhodium–hydride complex derived from [RhCl(cod)]_2_ seems to be suitable for the reactivity and selectivity in comparison with the corresponding rhodium–hydride complex from RhCl(PPh_3_)_3_ which has bulkier ligand.

**Scheme 4:**
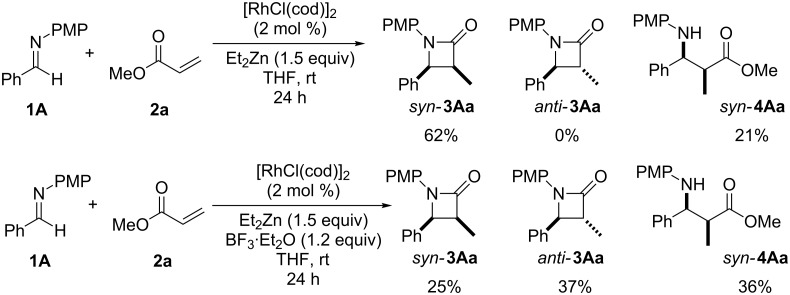
Effect of the Lewis acid addition.

**Figure 2:**
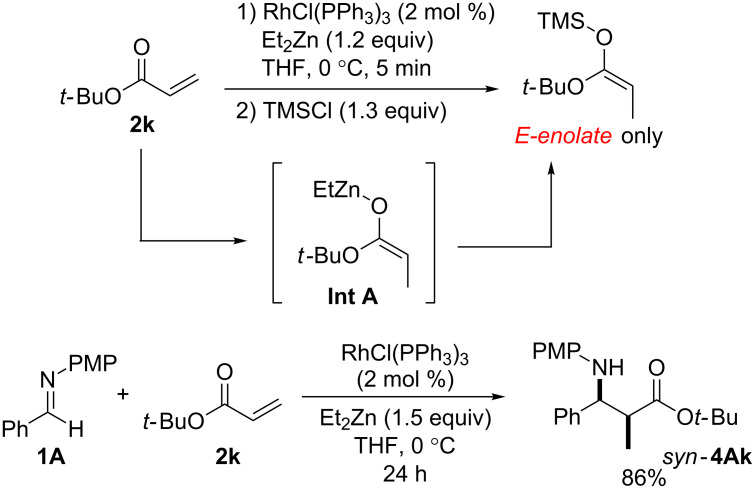
Reaction of **2k** and **1A** and the configuration of **Int A**.

The addition of a Lewis acid contributed not only to the activation the of imine but also the *anti* stereoselectivity of this reaction (Scheme 4). Furthermore, we reported in the previous paper that the configuration of the **Int A** species was identified as that of the *E*-enolate by the trapping procedure of *tert*-butyl acrylate (**2k**) with TMS-Cl. In addition, the same reaction gave only the *syn*-β-amino ester *syn*-**4Ak** in 86% and it was speculated that the reaction was proceeding via a linear transition state (Figure 2) [23].

**Scheme 5:**
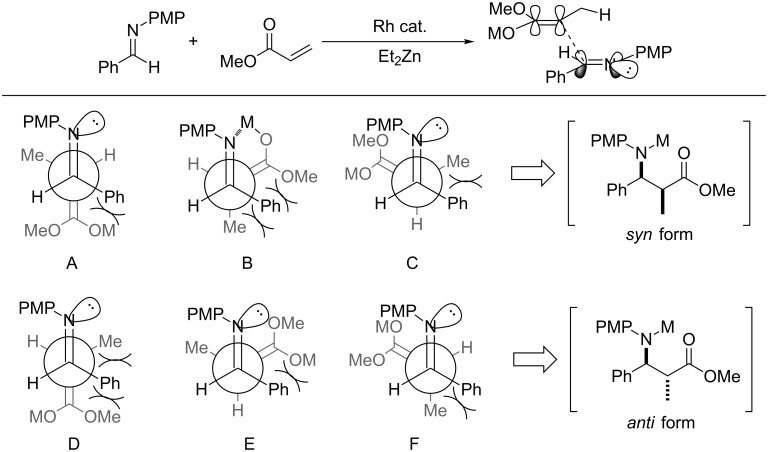
Transition-state model without Lewis acid.

**Scheme 6:**
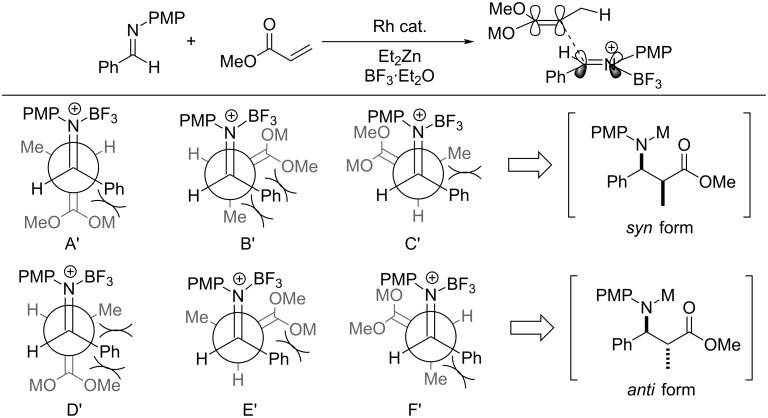
Transition-state model with Lewis acid.

When the reaction proceeded via a linear transition state, the reaction would be proposed to involve six competing transition state models (model A–F) as shown in Scheme 5. All these have steric repulsion but only the model B could be expected to have the interaction between the lone pair of the imine and the enolate metal. It seems the model B would be more stable than others to give the *syn* product. On the other hand, the addition of Lewis acid to the reaction mixture gave the iminium salt immediately and it would obstruct the stabilized metal interaction in model B’ of Scheme 6. This is meaning that the model F’ would be the most stable model, and the formation of the *anti* form product might increase relatively.

## Conclusion

In conclusion, we found improved conditions for reductive Mannich-type reaction using [RhCl(cod)]_2_ with Et_2_Zn, and the reaction proceeded smoothly to give various *syn*-β-lactams even if β-substituted α,β-unsaturated esters were employed as the substrates. In addition, we could also provide some perspectives on the reaction mechanism and the stereochemistry. When the reaction was carried out with imines and α,β-unsaturated lactones, similar β-lactams with *anti*-selectivities were obtained. Furthermore the yield and *anti* selectivity could be increased by adding Lewis acid, and we succeeded in achieving the effective synthesis of (±)-ezetimibe by using this reaction.

## Experimental

### General information

^1^H NMR and ^13^C NMR spectra were recorded on JNM-GX400 spectrometers and ECZS-400 spectrometers. ^19^F NMR spectra were recorded on Hitachi FT-NMR R-90H spectrometers. Chemical shifts of ^1^H NMR and ^13^C NMR are reported in ppm from tetramethylsilane (TMS) as an internal standard. Chemical shifts of ^19^F NMR are reported in ppm from benzotrifluoride (BTF) as an internal standard. All data are reported with the chemical shift(s), relative integration value, multiplicity (s = singlet, d = doublet, t = triplet, q = quartet, br = broad, m = multiplet), and coupling constants (Hz). Mass spectra were obtained on JEOL JMS-700T spectrometers. IR spectra were recorded on a Hitachi 270-30 infrared spectrophotometer. Melting points were measured on a Yanagimoto micro melting point apparatus MP-S3. Analytical gas–liquid chromatography (GLC) was carried out on a Hitachi G-3500 gas chromatograph (column; TC-5 0.25 mm × 15 m, carrier; He). Peak areas were calculated on a Hitachi D-2500 Chromato-Integrator.

### Materials

Tetrahydrofuran (THF) was purchased from Kanto Chemical Co. Inc. (Tokyo, Japan) in the dehydrated form. *N,N*-Dimethylformamide (DMF) and dichloromethane (CH_2_Cl_2_) were distilled over CaH_2_ and phosphorus pentoxide directly before use, respectively. All imines were prepared from corresponding aldehydes and amines. Methyl acrylate was distilled directly before use. Other commercially available reagents were used without further purification. All experiments were carried out under an argon atmosphere in flame-dried glassware using standard inert techniques for introducing reagents and solvents unless otherwise noted.

### General procedure for the *syn* selective synthesis of azetidin-2-one

In a manner closely related to a procedure in reference [23]: Imine (**1**) and α,β-unsaturated ester (**2**, 2 equiv) were added to a solution of 2 mol % of [RhCl(cod)]_2_ in DMF at 0 °C. Then, 1.0 M Et_2_Zn in hexane (3 equiv) was gradually added to the mixture at room temperature, and the mixture was stirred for 24 h. The mixture was quenched with sat. NH_4_Cl and extracted with AcOEt. The AcOEt layer was washed with sat. NaCl and dried over MgSO_4_. The solvent was removed in vacuo, and the residue was purified by column chromatography to give the corresponding *syn*-azetidin-2-one. The stereochemistry of the products were determined by the NOE between the CH_3_ and C_6_H_5_ groups on the azetidin-2-one ring and the coupling constant between each proton of C3 and C4 (*syn* form: *J* = 5.0–6.0 Hz, *anti* form: *J* = 2.0–3.0 Hz).

### General procedure for the *anti* selective synthesis of azetidin-2-one by the addition of BF_3_·Et_2_O

To a solution of [RhCl(cod)]_2_ (2 mol %) in DMF at room temperature was added imine (**1**). Then, BF_3_·Et_2_O (1.2 equiv) was added to the mixture and stirred for 30 min at the same temperature. Subsequently, 5,6-dihydro-2*H*-pyran-2-one (**2**, 1.2 equiv) was added to the mixture and then 1.0 M Et_2_Zn in hexane (3 equiv) was gradually added to the mixture at room temperature, and the mixture was stirred for 24 h. The mixture was quenched with sat. NH_4_Cl and extracted with AcOEt. The AcOEt layer was washed with sat. NaCl and dried over MgSO_4_. The solvent was removed in vacuo, and the residue was purified by column chromatography to give the corresponding *anti*-azetidin-2-one.

## Supporting Information

File 1 Experimental details, characterization of the compounds, X-ray crystallographic analysis of *anti*-**3Ai**, and ^1^H, ^13^C NMR spectra.
