# Supplementary material for: Rh-Catalyzed reductive Mannich-type reaction and its application towards the synthesis of (±)-ezetimibe
Source: Beilstein J Org Chem. 2016 Jul 27;12:1608–15. doi: 10.3762/bjoc.12.157 (PMC4979637; doi:10.3762/bjoc.12.157)

Rh-Catalyzed reductive Mannich-type reaction and its application towards the synthesis of (±)-ezetimibe

Motoyuki Isoda[1], Kazuyuki Sato[1], Yurika Kunugi[1], Satsuki Tokonishi[1], Atsushi Tarui[1], Masaaki Omote*[1], Hideki Minami[2] and Akira Ando*[1]

Address: [1]Faculty of Pharmaceutical Sciences, Setsunan University 45-1, Nagaotoge-cho, Hirakata, Osaka 573-0101, Japan and [2]Faculty of Pharmaceutical Sciences, Hiroshima International University, 5-1-1 Hirokoshingai, Kure, Hiroshima 737-0112, Japan

Email: Akira Ando* - aando@pharm.setsunan.ac.jp; Masaaki Omote* - omote@pharm.setsunan.ac.jp

*Corresponding author

Experimental details, characterization of the compounds, X-ray crystallographic analysis of *anti*-3Ai, and ^{1}H, ^{13}C NMR spectra

General information …………………………………..………………………. S2
Experimental section ………………………………………………………. S2
Materials …………………………………………………………………. S2
Typical procedure for the synthesis of *syn*-1-(*p*-methoxyphenyl)-3-methyl-4-phenylazetidin-2-one (*syn*-**3Aa**) …… S2
Typical procedure for the synthesis of *anti*-3-(3-hydroxypropyl)-1-(4-methoxyphenyl)-4-phenylazetidin-2-one (*anti*-**3Bh**)…… S3
Synthesis of 4-(4-(benzyloxy)phenyl)-1-(4-fluorophenyl)-3-(3-(4-fluorophenyl)-3-hydroxypropyl)azetidin-2-one (**3Bj**)…… S3
Synthesis of (±)-ezetimibe ………………………………………………. S4
Spectroscopic data ……………………….…………………………..... S4–S7
X-ray crystallographic analysis of *anti*-**4Ai**…..…………..…………... S8–S9
References ……………………………………………………………….. S10
^{1}H NMR and ^{13}C NMR charts………………………………..……S11–S18

General information:

^{1}H NMR and ^{13}C NMR spectra were recorded on JNM-GX400 spectrometers and ECZS-400 spectrometers. ^{19}F NMR spectra were recorded on Hitachi FT-NMR R-90H spectrometers. Chemical shifts of ^{1}H NMR and ^{13}C NMR are reported in ppm from tetramethylsilane (TMS) as an internal standard. Chemical shifts of ^{19}F NMR are reported in ppm from benzotrifluoride (BTF) as an internal standard. All data are reported with the chemical shift(s), relative integration value, multiplicity (s = singlet, d = doublet, t = triplet, q = quartet, br = broad, m = multiplet), and coupling constants (Hz). Mass spectra were obtained on JEOL JMS-700T spectrometers. IR spectra were recorded on a Hitachi 270-30 infrared spectrophotometer. Melting points were measured on a Yanagimoto micro melting point apparatus MP-S3. Analytical gas–liquid chromatography (GLC) was carried out on a Hitachi G-3500 gas chromatograph (column; TC-5 0.25 mm × 15 m, carrier; He). Peak areas were calculated on a Hitachi D-2500 Chromato-Integrator. ^{1}H and ^{13}C NMR spectra and characterization data for the compounds *syn*-/*anti*-**3Aa**, *syn*-**3Ad** and *anti*-**3Ah** are found in reference [1].

Experimental section:

Materials

Tetrahydrofuran (THF) was purchased from Kanto Chemical Co. Inc. (Tokyo, Japan) in the dehydrated form. *N*,*N*-Dimethylformamide (DMF) and dichloromethane (CH_2Cl_2) were distilled over CaH_2 and phosphorus pentoxide just before use, respectively. All imines were prepared from corresponding aldehydes and amines. Methyl acrylate was distilled just before use. Other commercially available reagents were used without further purification. All experiments were carried out under an argon atmosphere in flame-dried glassware using standard inert techniques for introducing reagents and solvents unless otherwise noted.

Typical procedure for the synthesis of *syn*-1-(*p*-methoxyphenyl)-3-methyl-4-phenyl-azetidin-2-one (*syn*-3Aa)

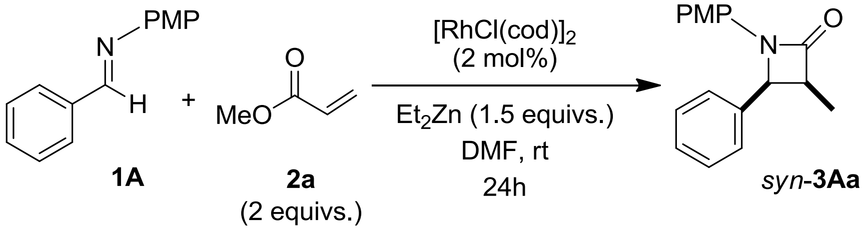

In a manner closely related to a procedure in reference [1]: (*E*)-*N*-benzylidene-4-methoxy-benzenamine (**1A**, 0.5 mmol) and methyl acrylate (**2a**, 1 mmol) was added to a solution of $[RhCl(cod)]_2$ (2 mol %) in DMF (1.25 mL) at 0 °C. Then, 1.0 M Et_2Zn in hexane (1.5 mmol, 1.5 mL) was gradually added to the mixture at room temperature, and the mixture was stirred

at same temperature for 24 h. The mixture was quenched with sat. NH_4Cl and extracted with AcOEt. The AcOEt layer was washed with sat. NaCl and dried over $MgSO_4$. The solvent was removed in vacuo, and the residue was purified by column chromatography (AcOEt/hexane = 2:8) and the diastereomers were obtained in 69% (92.4 mg; *syn* form) and 9% yield (11.5 mg; *anti* form), respectively. The stereochemistry of products were determined by NOE between the CH_3 and C_6H_5 groups on azetidin-2-one ring and coupling constant between each protons of C3 and C4 (*syn* form: J = 5.0–6.0 Hz, *anti* form: J = 2.0–3.0 Hz) [2].

Typical procedure for the synthesis of *anti*-3-(3-hydroxypropyl)-1-(4-methoxyphenyl)-4-phenylazetidin-2-one (*anti*-3Bh)

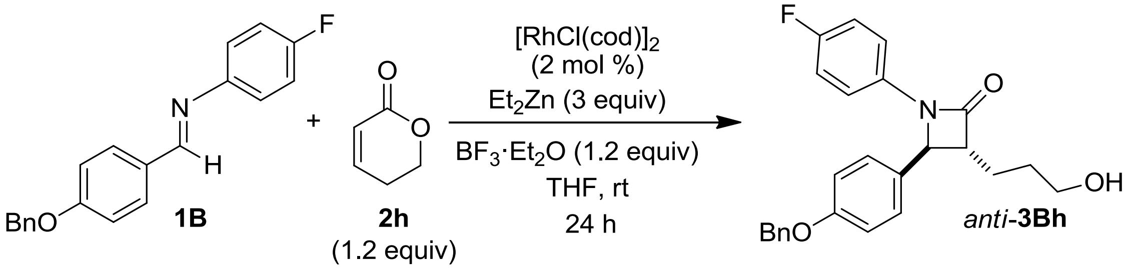

To a solution of $[RhCl(cod)]_2$ (2 mol %) and *N*-(4-(benzyloxy)benzylidene)-4-fluoroaniline (**1B**, 0.5 mmol) in THF (1.25 mL) at room temperature was added $BF_3{\cdot}Et_2O$ (0.6 mmol), and then it stirred for 30 min at the same temperature. Subsequently, 5,6-dihydro-2*H*-pyran-2-one (**2h**, 0.6 mmol) was added to the suspension mixture, and then 1.0 M Et_2Zn in hexane (1.5 mmol, 1.5 mL) was gradually added to the mixture and stirred for 24 h. The mixture was quenched with sat. NH_4Cl and extracted with AcOEt. The AcOEt layer was washed with sat. NaCl and dried over $MgSO_4$. The solvent was removed in vacuo, and the residue was purified by column chromatography (AcOEt/hexane = 4:6) and *anti* diastereomer was obtained in 46% (93.3 mg) [2].

Synthesis of 4-(4-(benzyloxy)phenyl)-1-(4-fluorophenyl)-3-(3-(4-fluorophenyl)-3-hydroxypropyl)azetidin-2-one (3Bj)

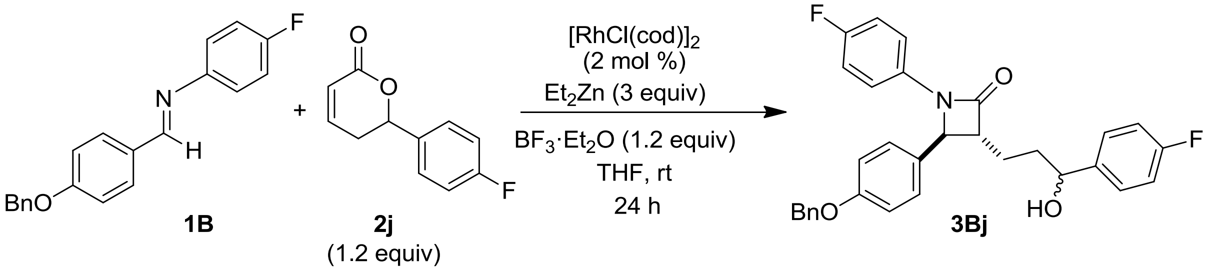

To a solution of $[RhCl(cod)]_2$ (2 mol %) and *N*-(4-(benzyloxy)benzylidene)-4-fluoroaniline (**1B**, 0.5 mmol) in THF (1.25 mL) at room temperature was added $BF_3{\cdot}Et_2O$ (0.6 mmol) was added to the mixture, and then it stirred for 30 min at the same temperature. Subsequently, 6-(4-fluorophenyl)-5,6-dihydro-2*H*-pyran-2-one (**2j**, 0.6 mmol) was added to the suspension

mixture, and then 1.0 M Et_2Zn in hexane (1.5 mmol, 1.5 mL) was gradually added to the mixture and stirred for 24 h. The mixture was quenched with sat. NH_4Cl and extracted with AcOEt. The AcOEt layer was washed with sat. NaCl and dried over $MgSO_4$. The solvent was removed in vacuo, and the residue was purified by column chromatography (AcOEt/hexane = 5:5) to give **3Bj** in 58% (144.9 mg).

Synthesis of (±)-ezetimibe

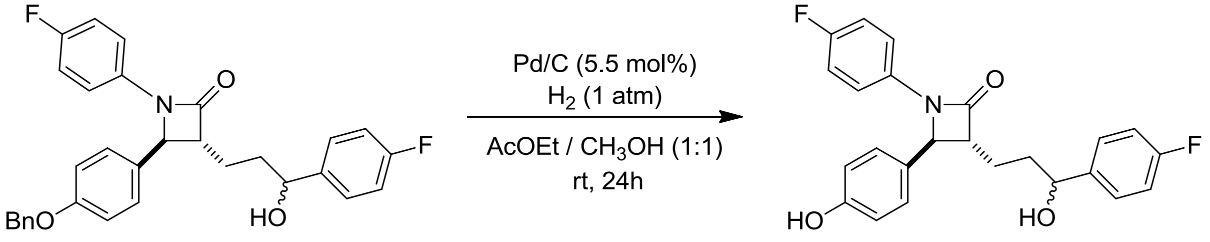

3Bj **(±)-Ezetimibe**

To a suspension of 10% Pd/C (5.5 mol %) in 1:1 AcOEt/CH_3OH (10 mL) was added **3Bj** (0.2 mmol), and then it placed under H_2 (1 atm) for 24 h. Then the mixture was filtered, and the solvent was removed in vacuo. The residue was purified by column chromatography (AcOEt/hexane = 5:5) to give (±)-ezetimibe in 80% (65.5 mg).

Spectroscopic Data:

3-Benzyl-1-(4-methoxyphenyl)-4-phenylazetidin-2-one (3Ab)

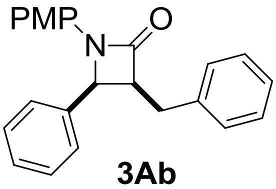

3Ab

The title product (**3Ab**) was purified by column chromatography (AcOEt/hexane = 2:8) and only the *syn* diastereomer was obtained in 66% yield (113.5 mg).

syn-**3Ab**: A colorless solid; M.p. 140.0–140.5 °C; ^{1}H NMR ($CDCl_3$) δ: 2.46 (1H, dd, J = 15.2, 10.0 Hz), 2.98 (1H, dd, J = 15.2, 5.2 Hz), 3.73 (3H, s), 3.90 (1H, dt, J = 15.2, 5.2 Hz), 5.15 (1H, d, J = 5.2 Hz), 6.74–6.80 (4H, m), 7.10–7.18 (4H, m), 7.22–7.26 (3H, m), 7.30–7.35 (3H, m); ^{13}C NMR (100 MHz, $CDCl_3$) δ: 31.1, 55.5, 55.7, 58.7, 114.4, 118.5, 126.2, 127.6, 128.3, 128.5, 128.6, 128.7, 131.2, 134.8, 138.4, 156.0, 166.7; MS *m/z*: 343 (M^+); HRMS Calcd. for $C_{23}H_{21}NO_2$: 343.157 (M^+), Found: 343.157; IR (KBr) cm^{-1}:1722.

1-(4-Methoxyphenyl)-3,3-dimethyl-4-phenylazetidin-2-one (3Ae)

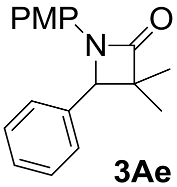

3Ae

The title product (**3Ae**) was purified by column chromatography (AcOEt/hexane = 2:8) and was obtained in 98% yield (137.2 mg).

3Ae: A colorless solid; M.p. 146.5–147.0 °C; ^{1}H NMR ($CDCl_3$) δ: 0.83 (3H, s), 1.51 (3H, s), 3.14 (3H, s), 4.76 (1H, s), 6.77–6.81 (2H, m), 7.17–7.20 (2H, m), 7.23–7.36 (4H, m); ^{13}C NMR (100 MHz, $CDCl_3$) δ: 18.1, 22.9, 55.5, 55.6,

66.7, 114.4, 118.6, 126.7, 128.1, 128.7, 131.6, 135.8, 155.9, 171.0 ; MS *m/z*: 281 (M^+); HRMS Calcd. for $C_{18}H_{19}NO_2$: 281.142 (M^+), Found: 281.142; IR (KBr) cm^{-1}:1731.

3-(But-1-enyl)-1-(4-methoxyphenyl)-4-phenylazetidin-2-one (3Ag)

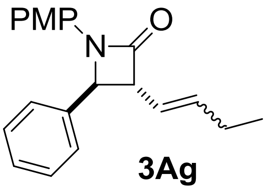

3Ag

The title product (**3Ag**) was purified by column chromatography (AcOEt/hexane = 2:8) and was obtained in 59% yield (90.0 mg) as *anti* product of (*E*/*Z*)-mixture.

(E/Z)-anti-**3Ag**: A colorless viscous oil; ^{1}H NMR ($CDCl_3$) δ: 0.93 (0.3H, t, *J* = 7.2 Hz), 1.01 (3H, t, *J* = 7.2), 1.92–2.04 (0.2H, m), 2.06–2.13 (2H, m), 3.64–3.67 (1H, m), 3.73 (1.1H, m), 3.93–3.96 (0.1H, m), 4.67 (0.1H, d, *J* = 2.8 Hz), 4.70 (1H, d, *J* = 2.4 Hz), 5.57–5.66 (1.1H, m), 5.70–5.84 (1.1H, m), 6.75–6.79 (2.2H, m), 7.20–7.25 (2.2H, m), 7.29–7.38 (5.5H, m); ^{13}C NMR (100 MHz, $CDCl_3$) δ: 13.3, 14.3, 21.5, 22.7, 25.7, 55.1, 59.3, 62.0, 62.2, 63.6, 114.4, 118.38, 118.43, 121.3, 121.4, 125.9, 126.0, 128.5, 129.2, 131.4, 137.8, 138.1, 138.2, 156.1, 165.8; MS *m/z*: 307 (M^+); HRMS Calcd. for $C_{20}H_{21}NO_2$: 307.157 (M^+), Found: 307.157.

3-(4-Methoxyphenylamino)-*N*,*N*,2-trimethyl-3-phenylpropanamide (4Ai)

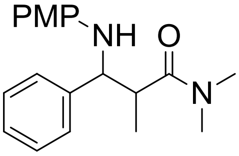

4Ai

The title product (**4Ai**) was purified by column chromatography (AcOEt/hexane = 4:6) and each diastereomers were obtained in 5% (7.8 mg; *syn* form) and 22% yield (34.9 mg; *anti* form), respectively.

syn-**4Ai**: A colorless solid; M.p. 180.0–182.0; ^{1}H NMR ($CDCl_3$) δ: 1.34 (3H, d, *J* = 6.8 Hz), 2.37 (3H, s), 2.78 (3H, s), 3.20–3.26 (1H, m), 3.66 (3H, s), 4.42 (1H, d, *J* = 4.0 Hz), 5.65 (1H, br), 6.41–6.45 (2H, m), 6.62–7.66 (2H, m), 7.17–7.22 (1H, m), 7.24–7.30 (4H, m); ^{13}C NMR (100 MHz, $CDCl_3$) δ: 16.9, 35.5, 37.1, 41.1, 55.9, 61.9, 113.9, 114.9, 126.5, 127.3, 128.6, 141.8, 143.2, 151.3, 174.6; MS *m/z*: 312 (M^+); HRMS Calcd. for $C_{19}H_{24}N_2O_2$: 312.184 (M^+), Found: 312.184; IR (KBr) cm^{-1}: 3357, 1633.

anti-**4Ai**: A colorless solid; M.p. 119.5–120.5 °C; ^{1}H NMR ($CDCl_3$) δ: 1.14 (3H, d, *J* = 6.8 Hz), 1.56 (1H, s), 2.86 (3H, s), 2.95 (3H, s), 3.07–3.13 (1H, m), 3.66 (3H, s), 4.40 (1H, d, *J* = 5.2 Hz), 4.64 (1H, br), 6.41–6.45 (2H, m), 6.62–6.66 (2H, m), 7.20–7.40 (5H, m); ^{13}C NMR (100 MHz, $CDCl_3$) δ: 12.4, 35.8, 37.4, 42.1, 55.8, 60.6, 114.7, 115.2, 126.9, 127.3, 128.6, 142.1, 142.1, 152.1, 174.7; MS *m/z*: 312 (M^+); HRMS Calcd. for $C_{19}H_{24}N_2O_2$: 312.184 (M^+), Found 312.184; IR (KBr) cm^{-1}: 3350, 1633.

3-(3-Hydroxypropyl)-1-(*p*-methoxyphenyl)-4-phenylazetidin-2-one (3Bh)

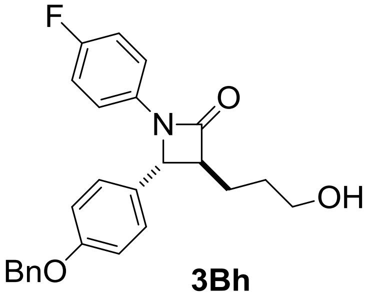

3Bh

The title product (**3Bh**) was purified by column chromatography (AcOEt/hexane = 4:6) and only the *anti* diastereomer was obtained in 46% (93.3 mg).

anti-**3Bh**: A colorless solid; M.p. 75.5–76.0 °C; ^{1}H NMR ($CDCl_3$) δ: 1.70–2.05 (5H, m), 3.10 (1H, td, *J* = 8.0, 2.4 Hz), 3.68 (2H, t, *J* = 6.4 Hz), 4.56 (1H, d, *J* = 2.4 Hz), 5.04 (2H, s), 6.89–6.98 (4H, m), 7.21–7.27 (4H, m), 7.30–7.42 (5H, m); ^{13}C NMR (100 MHz, $CDCl_3$) δ:25.3, 30.4, 60.5, 61.3, 62.2, 70.2, 115.6, 115.9 (d, *J* = 9 Hz), 118.5 (d, *J* = 22 Hz), 127.3, 127.6, 128.2, 128.7, 129.7, 134.0, 136.7, 159.1 (d, *J* = 243 Hz), 159.2, 168.0; ^{19}F NMR ($CDCl_3$, 376 MHz) δ: -55.4 (1F, m); MS *m*/*z*: 405 (M^+); HRMS Calcd. for $C_{25}H_{24}FNO_3$: 405.174 (M^+), Found: 405.174; IR (KBr) cm^{-1}: 3392, 1732.

4-(4-(Benzyloxy)phenyl)-1-(4-fluorophenyl)-3-(3-(4-fluorophenyl)-3-hydroxypropyl)azetidin-2-one (3Bj)

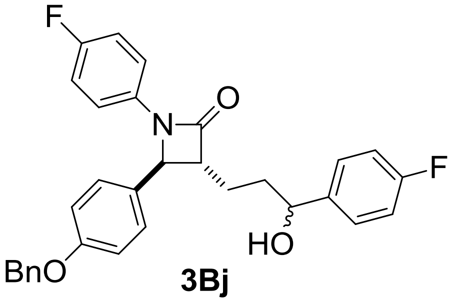

3Bj

The title product (**3Bj**) was purified by column chromatography (AcOEt/hexane = 5:5) to give *anti*-**3Bj** in 58% (144.9 mg) as a diastereomeric mixture.

anti-**3Bj**: A colorless oil; ^{1}H NMR ($CDCl_3$) δ: 1.79–2.06 (4H, m), 2.31 (1H, br), 3.04–3.13 (1H, m), 4.51–4.57 (1H, m), 4.70 (1H, m), 5.04 (2H, s), 6.88–7.04 (6H, m), 7.19–7.42 (11H, m); ^{13}C NMR (100 MHz, $CDCl_3$) δ: 25.1, 36.6, 36.7, 60.4, 60.4, 61.2, 70.2, 73.2, 73.4, 115.4 (d, *J* = 22.1 Hz), 115.5 (d, *J* = 21.1 Hz), 115.6, 115.9 (d, *J* = 23.0 Hz), 118.5 (d, *J* = 8.5 Hz), 127.3, 127.4 (d, *J* = 22.0 Hz), 127.5 (d, *J* = 7.7 Hz), 127.6, 128.2, 128.7, 129.7, 134.0, 136.7, 140.0 (d, *J* = 2.9 Hz), 140.1 (d, *J* = 2.9 Hz), 159.0 (d, *J* = 242.4 Hz), 159.1, 162.3 (d, *J* = 244.4 Hz), 167.7, 167.8; ^{19}F NMR ($CDCl_3$, 376 MHz) δ: -51.7–-51.8 (1F, m), -54.9–-55.0 (1F, m); MS *m*/*z*: 499 (M^+); HRMS Calcd. for $C_{31}H_{27}F_2NO_3$: 499.196 (M^+), Found: 499.196.

***anti*-1-(4-Fluorophenyl)-3-(3-(4-fluorophenyl)-3-hydroxypropyl)-4-(4-hydroxyphenyl)azetidin-2-one [(±)-Ezetimibe]**

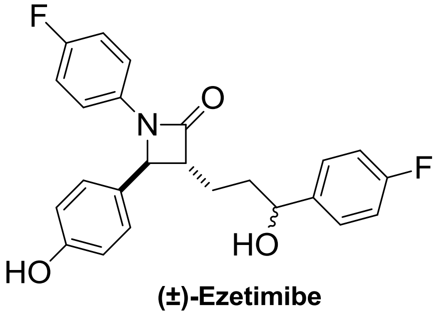

(±)-Ezetimibe

The title product (**(±)-ezetimibe**) was purified by column chromatography (AcOEt/hexane = 5:5) to give **(±)-ezetimibe** in 80% (65.5 mg) as a diastereomeric mixture.

(±)-ezetimibe: A colorless solid; ^{1}H NMR (DMSO-d6) δ: 1.59–1.86 (4H, m), 3.00–3.06 (1H, m), 4.42–4.53 (1H, m), 4.75–4.77 (1H, m), 5.23–5.24 (1H, m), 6.69–6.72 (2H, m),

7.05–7.13 (4H, m), 7.14–7.20 (4H, m), 7.23–7.31 (2H, m), 9.48 (1H, br); ^{13}C NMR (100 MHz, DMSO-d6) δ: 25.1, 36.9, 59.9, 60.0, 60.1, 71.6, 71.7, 115.2 (d, *J* = 20.2 Hz), 116.2, 116.5, 118.8 (d, *J* = 7.5 Hz), 128.1 (d, *J* = 13.4 Hz), 128.1, 128.4 (d, *J* = 4.8 Hz), 134.5, 142.6 (d, *J* = 4.8 Hz), 142.7 (d, *J* = 5.7 Hz), 158.0, 158.5 (d, *J* = 238.6 Hz), 161.6 (d, *J* = 240.6 Hz), 167.8, 167.9; ^{19}F NMR ($CDCl_3$, 376 MHz) δ: -55.4–-55.5 (1F, m), -57.7–-57.8 (1F, m); MS *m*/*z*: 409 (M^+); HRMS Calcd. for $C_{24}H_{21}FNO_3$: 409.149 (M^+), Found: 409.149.

X-ray crystallographic analysis of *anti*-4Ai:

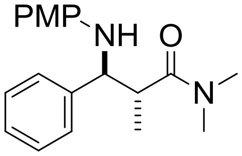

4Ai

The product was recrystallized from ethyl acetate/hexane. The single crystal was mounted on a glass fiber. All measurements were made on a Rigaku R-AXIS RAPID diffractometer using graphite monochromated CuKα (λ = 1.54187 Å) radiation. The 2θ (max) value cut of 136.5°. The crystal structure was solved by the SIR2008 [3] direct methods and refined by the full-matrix least squares using the program CRYSTALS [4]. The crystallographic data were summarized in the following table.

emprical formula	$C_{19}H_{24}N_2O_2$
formula weight	312.41
crystal system	monoclinic
space group	P 21/c
a, Å	12.6178(3)
b, Å	5.97519(16)
c, Å	23.2562(6)
β, °	101.815(7)
V, Å^3	1716.23(9)
Z	4
D_{calc}, g/cm^3	1.209
T, °C	23
μ (CuKα), cm^{-1}	6.25
No. of reflens measured	17797
No. of reflens observed	2672
No. of reflens variable	232
R (All reflections)	0.0459
R_w (All reflections)	0.0571
Good of Fit	1.364
Max Shift/Error	0.001

The crystal structure has been deposited at the Cambridge Crystallographic Data Centre (CCDC 1431128). The data can be obtained free of charge via the Internet at www.ccdc.cam.ac.uk/conts/retrieving.html.

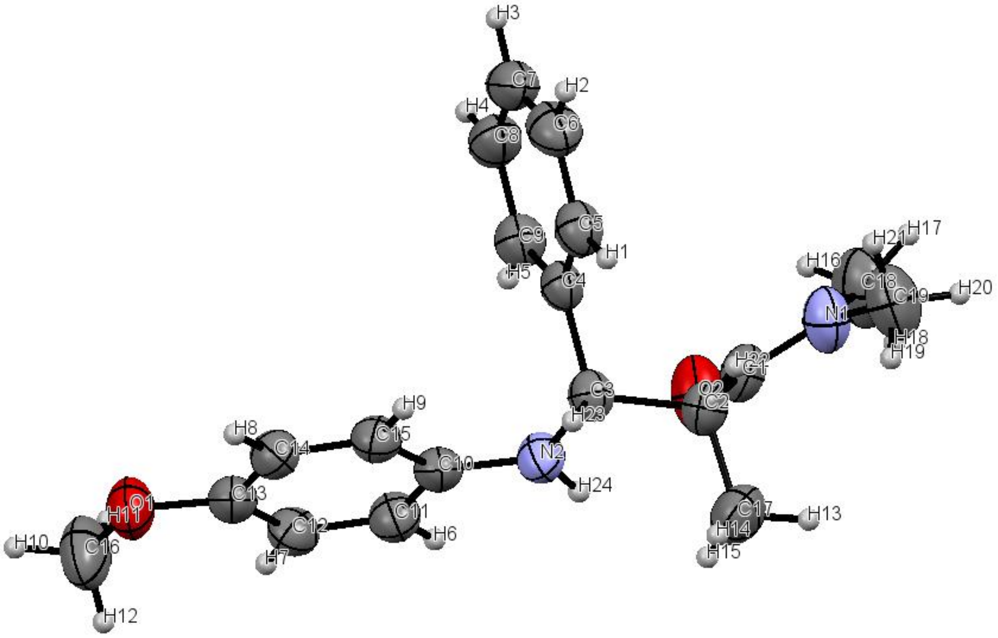
H3
C7
H2
H4
C6
C8
C5
C9
H1
H5
C4
H16
H21
H17
C18
C19
H20
N1
H18
H19
C1
O2
C3
C2
H9
H23
H8
C15
C14
C10
N2
H24
O1
C13
C11
H11
C17
H13
H10
C16
C12
H6
H14
H7
H15
H12

References:

1) Isoda, M.; Sato, K.; Funakoshi, M.; Omote, M.; Tarui, A.; Omote, M.; Ando, A. *J. Org. Chem*. **2015**, *80*, 8398–8405.
2) Sato, K.; Isoda, M.; Ohata, S.; Morita, S.; Tarui, A.; Omote, M.; Kumadaki, I.; Ando, A. *Adv. Synth. Catal.* **2012**, *35*4, 510–514.
3) SIR2008: M.C. Burla, R. Caliandro, M. Camalli, B. Carrozzini, G.L. Cascarano, L. De Caro, C. Giacovazzo, G. Polidori, D. Siliqi, R. Spagna (2007).
4) Carruthers, J.R., Rollett, J.S., Betteridge, P.W., Kinna, D., Pearce, L., Larsen, A., and Gabe, E. (1999) CRYSTALS Issue 11, Chemical Crystallography Laboratory, Oxford, UK.

syn-**3Ab**

^{1}H NMR

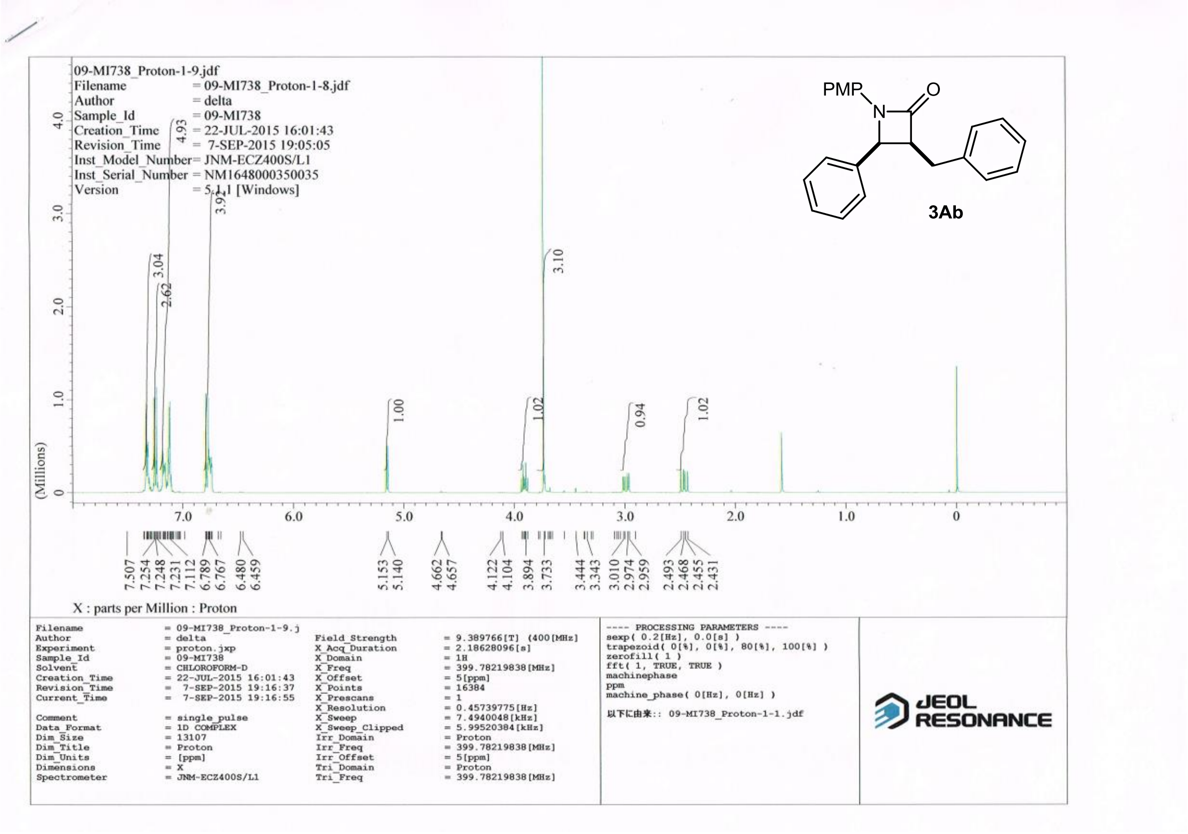

^{13}C NMR

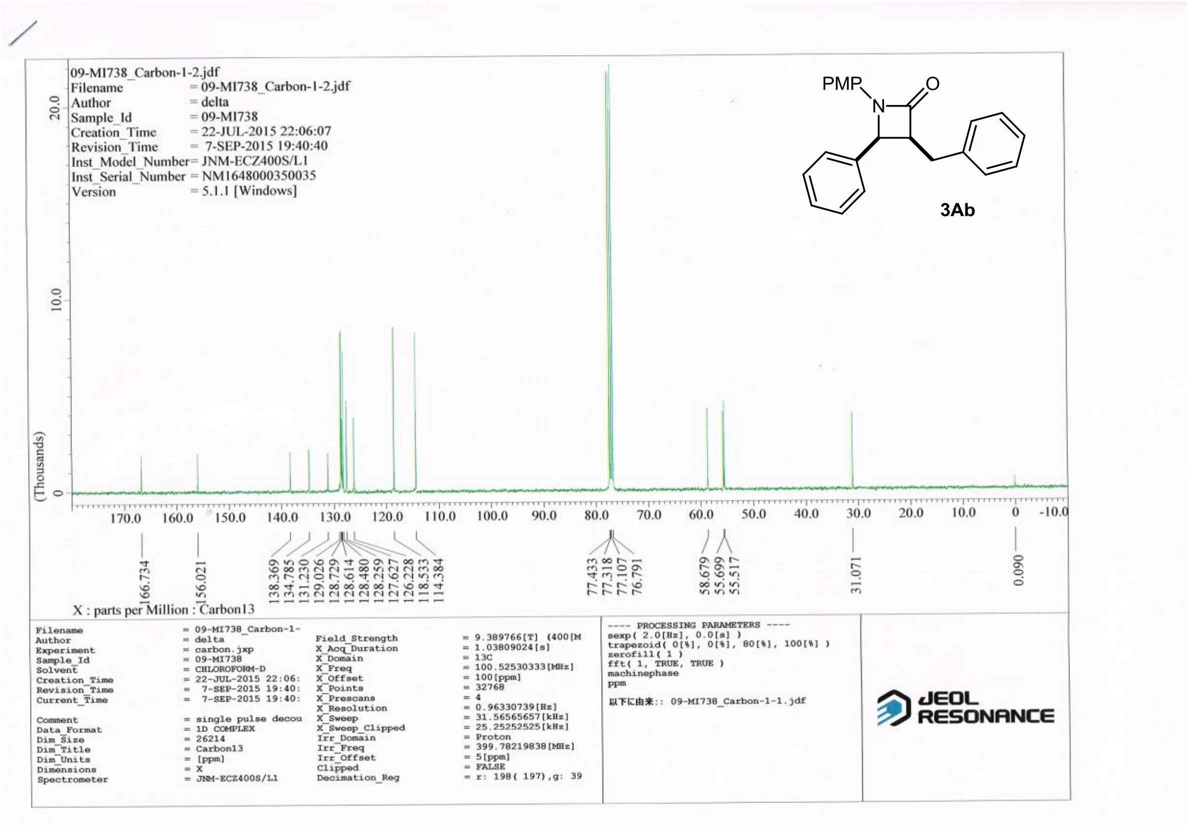

syn-**3Ae**

^{1}H NMR

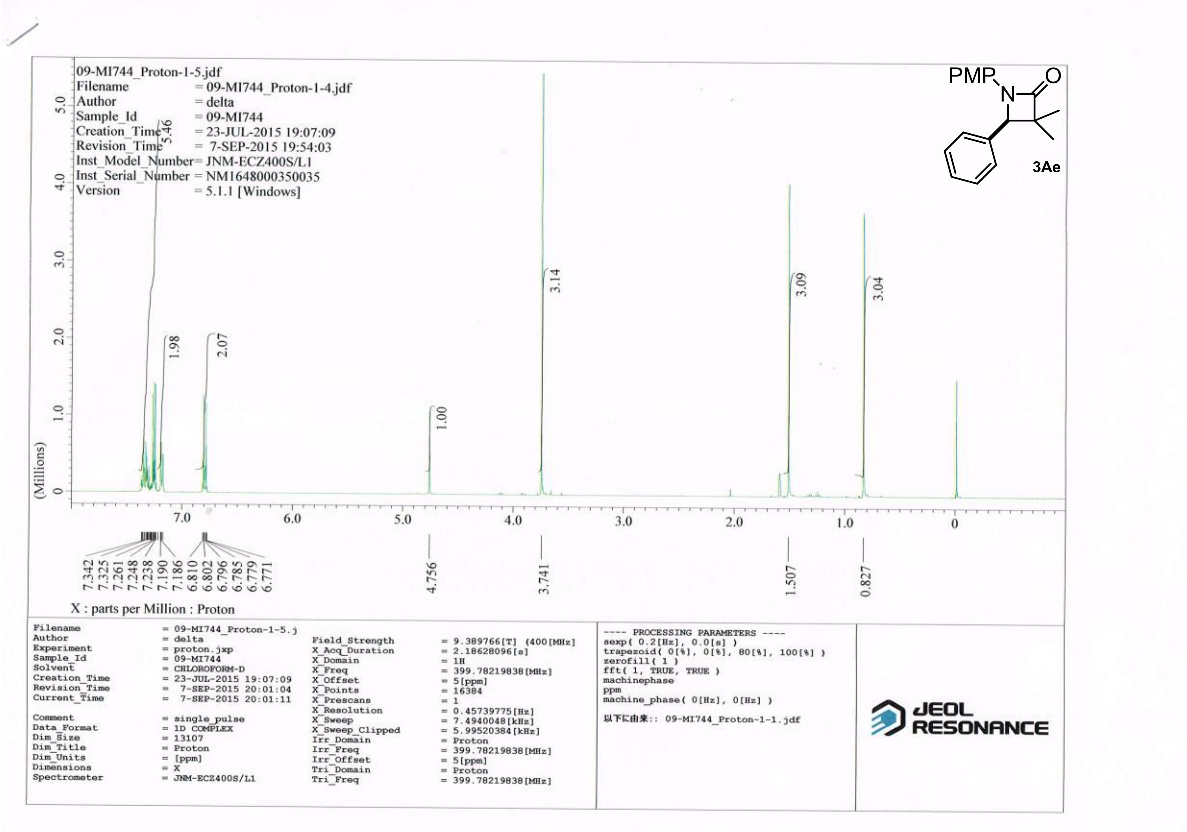

^{13}C NMR

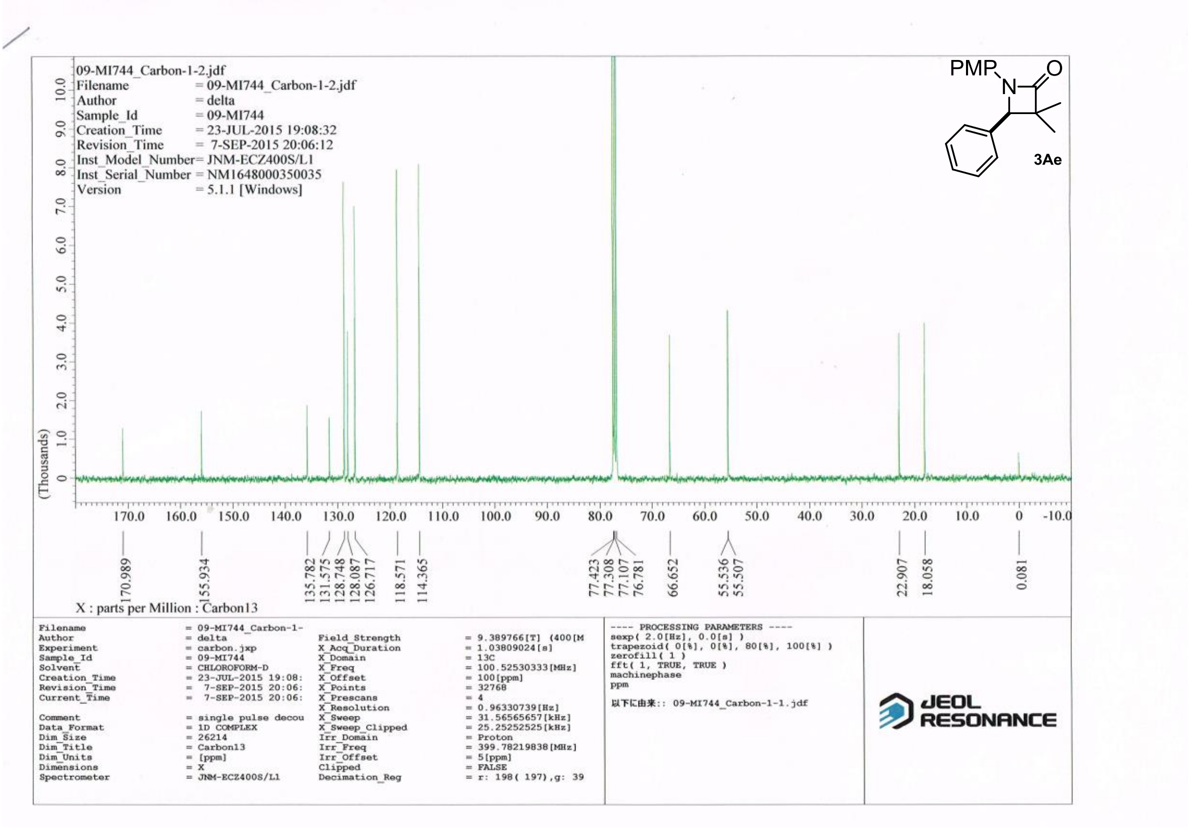

anti-**3Ag**

^{1}H NMR

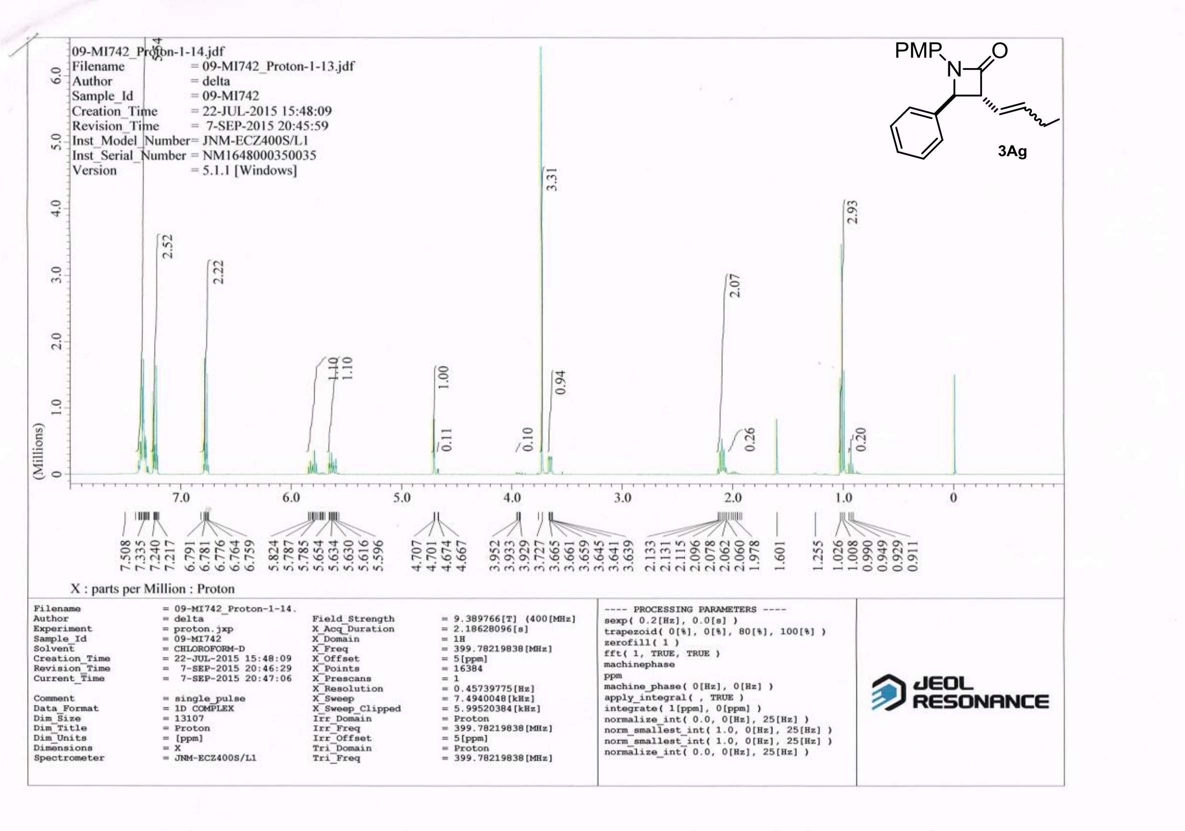

^{13}C NMR

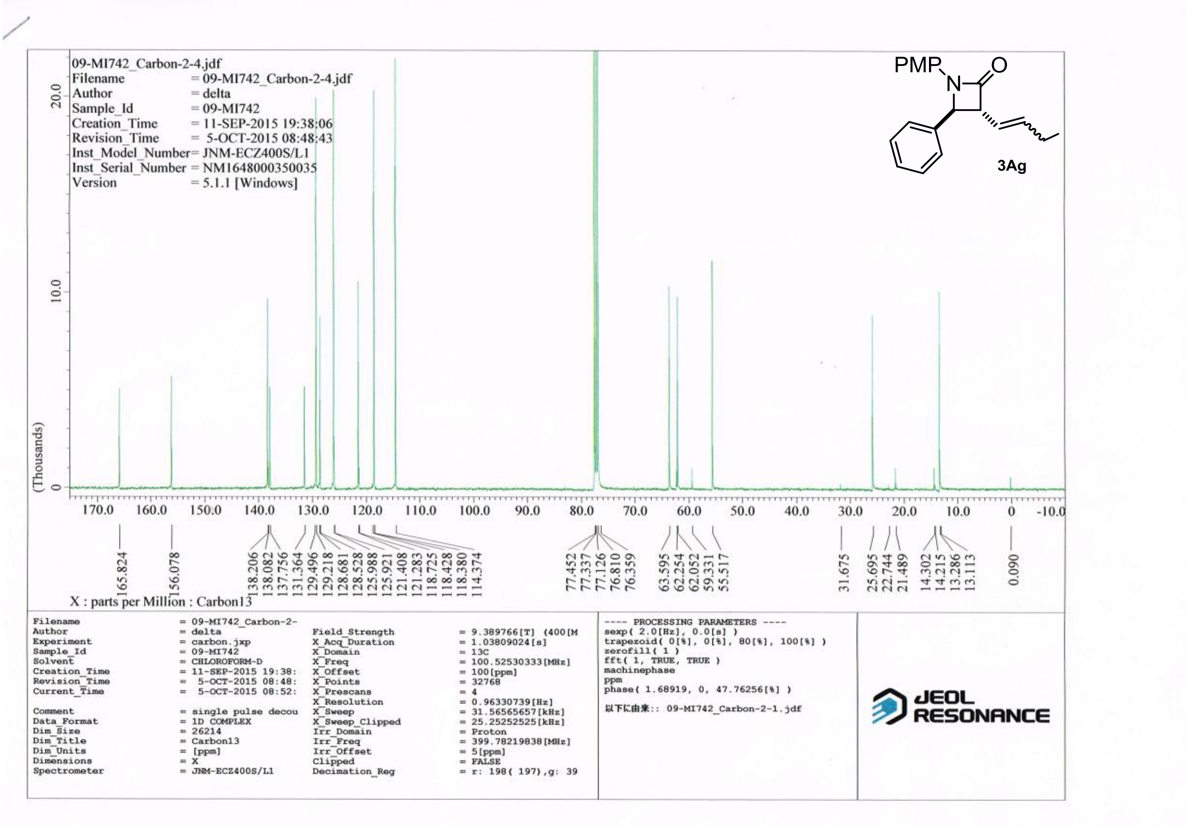

syn-**4Ai**

^{1}H NMR

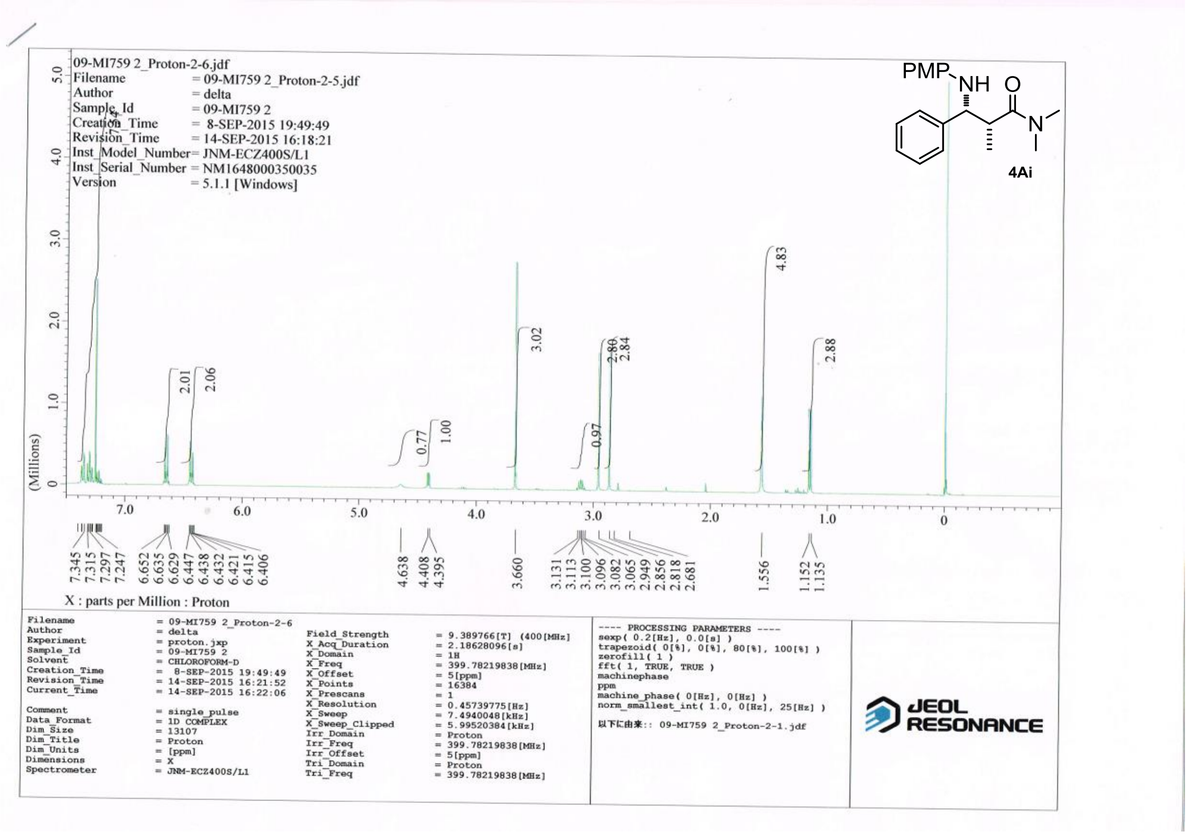

^{13}C NMR

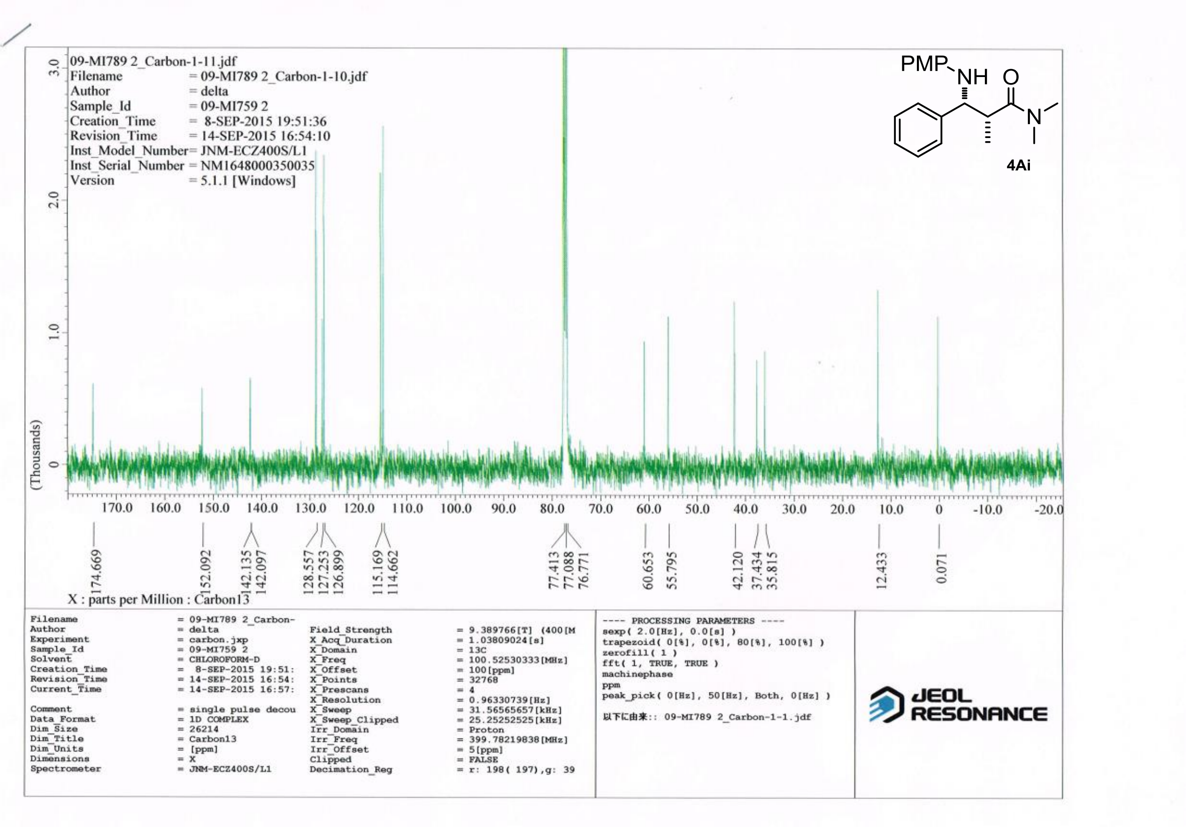

anti-**4Ai**

^{1}H NMR

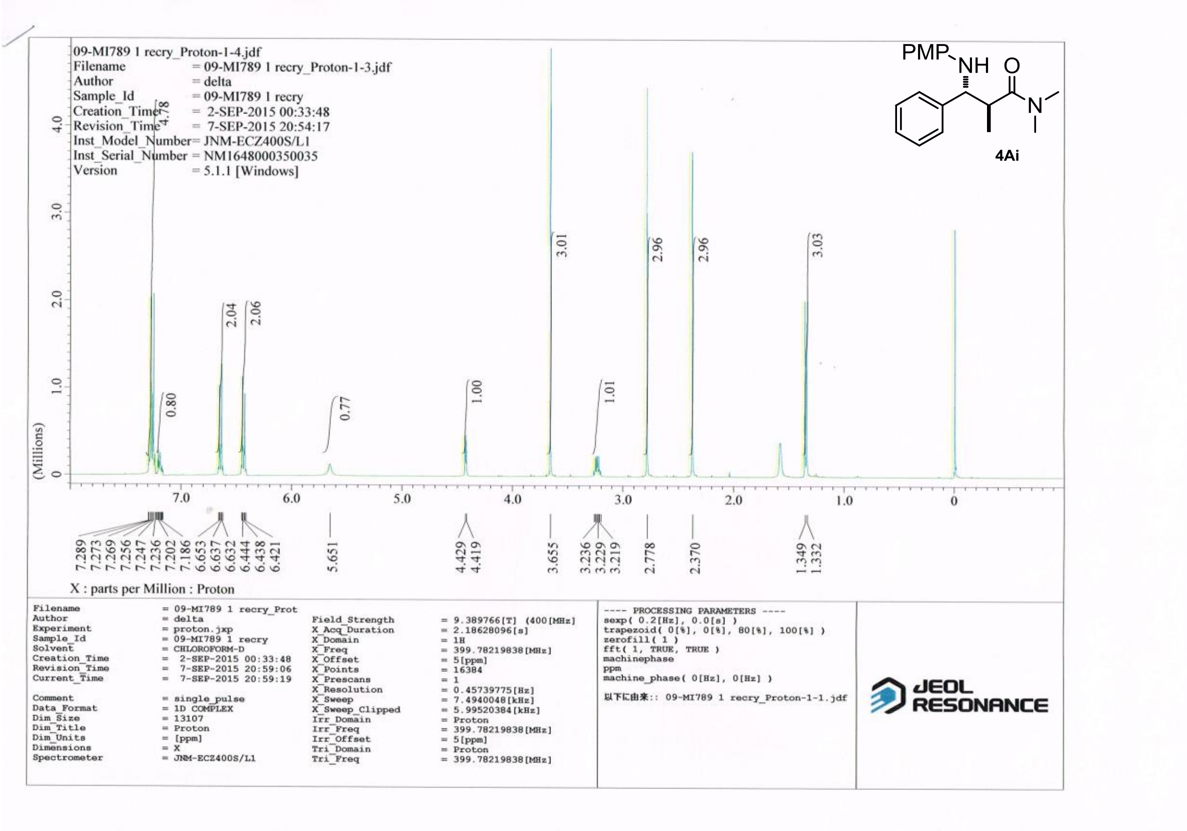

^{13}C NMR

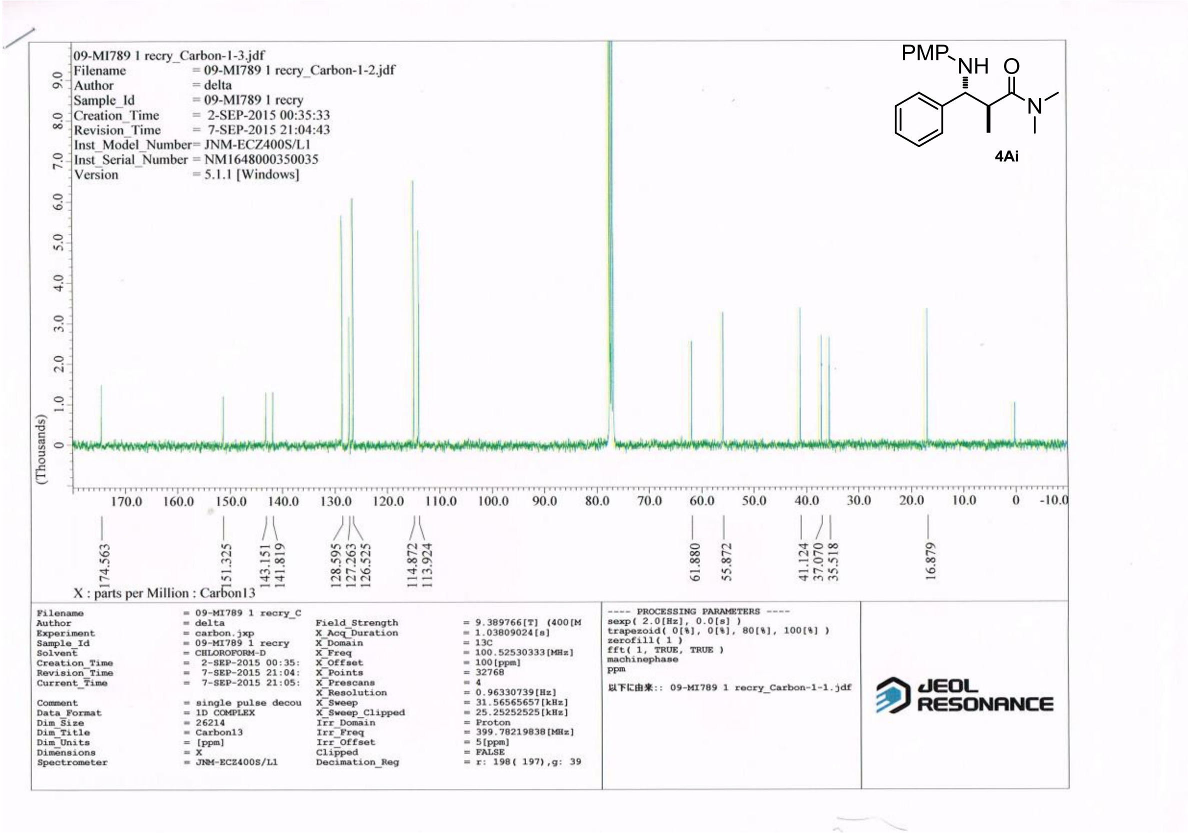

anti-**3Bh**

^{1}H NMR

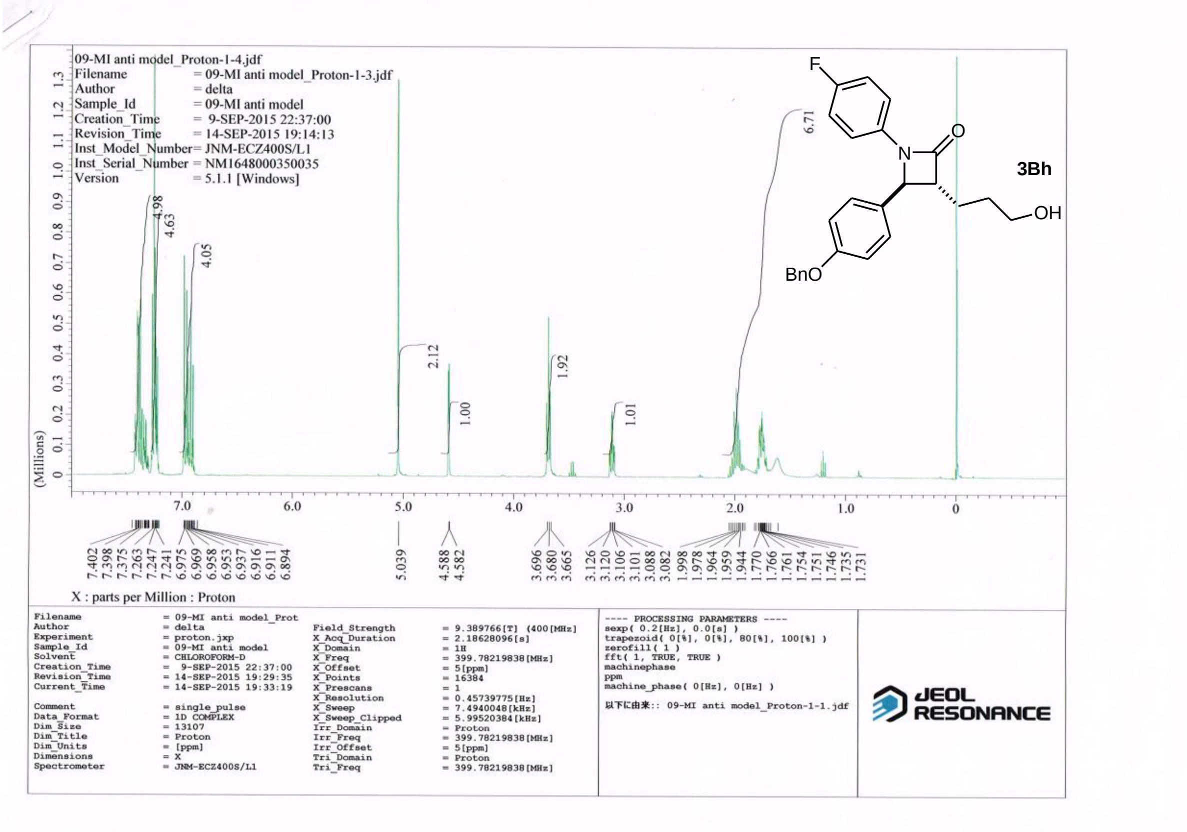

^{13}C NMR

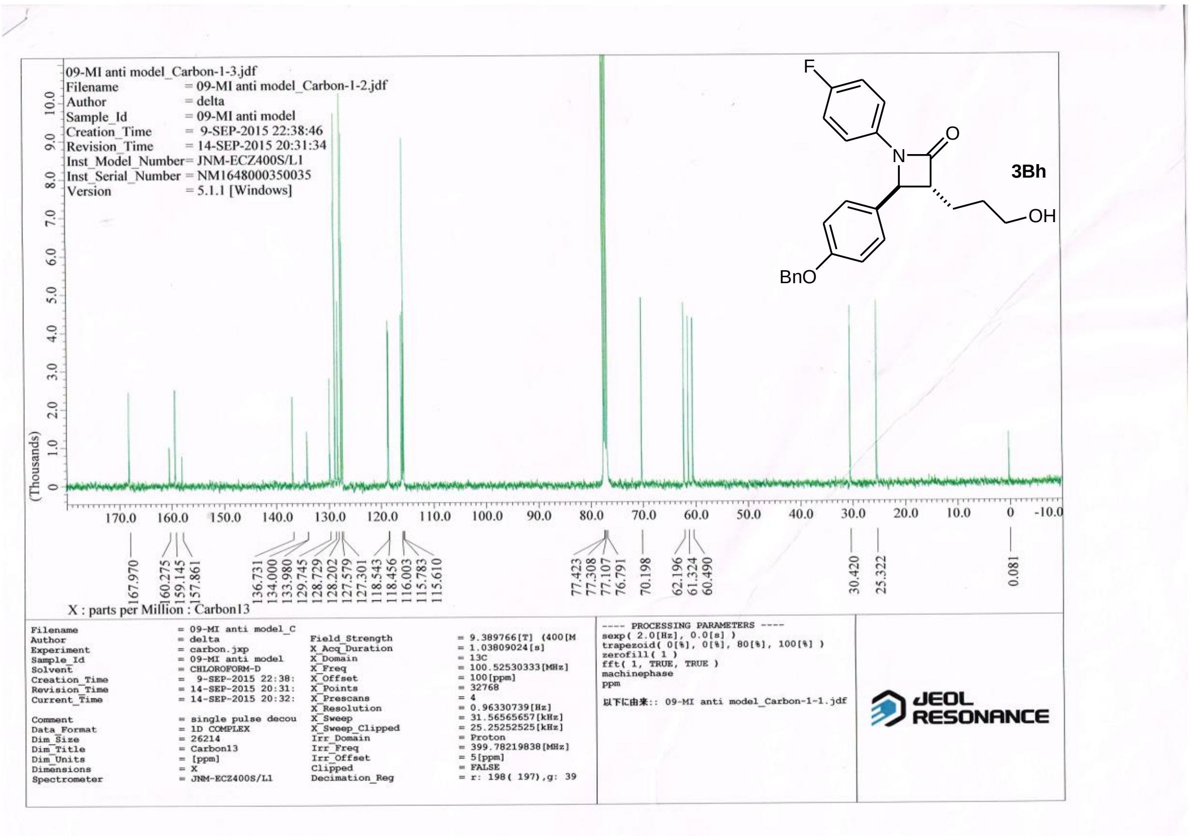

anti-**3Bj**

^{1}H NMR

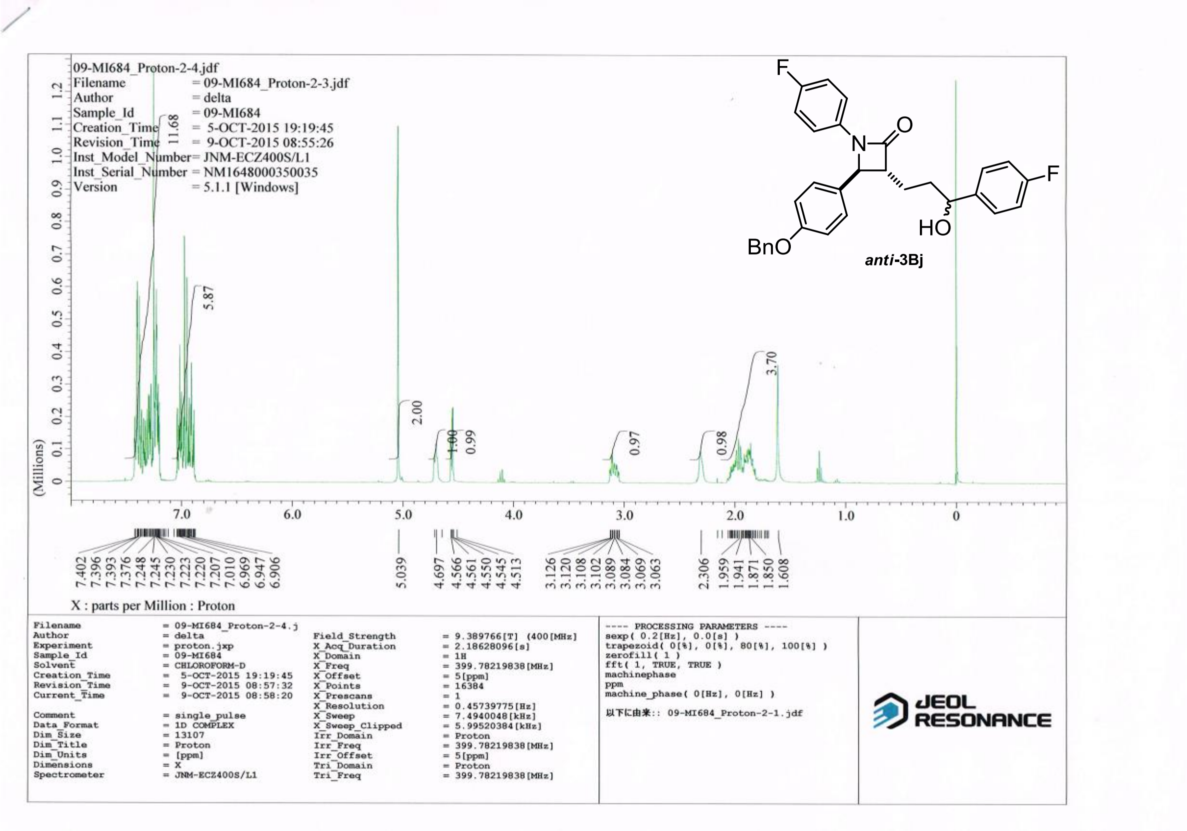

^{13}C NMR

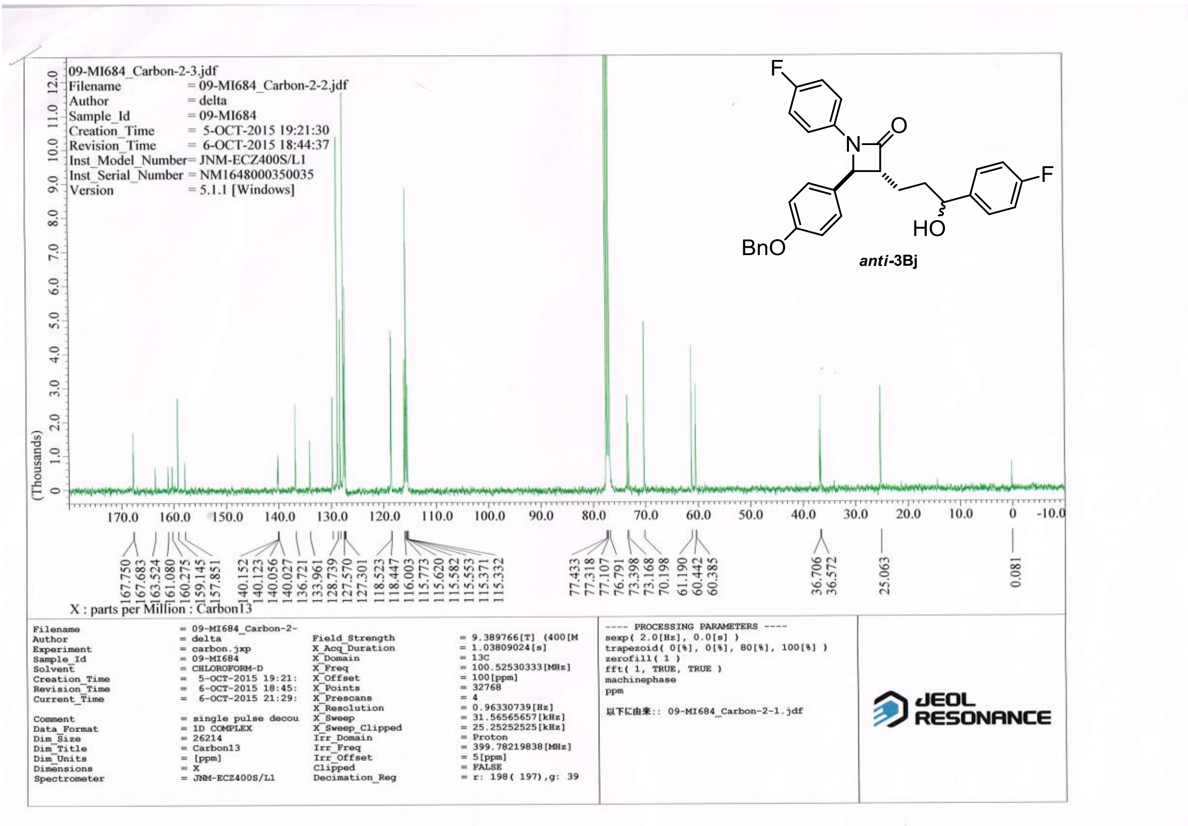

(±)-Ezetimibe

^{1}H NMR

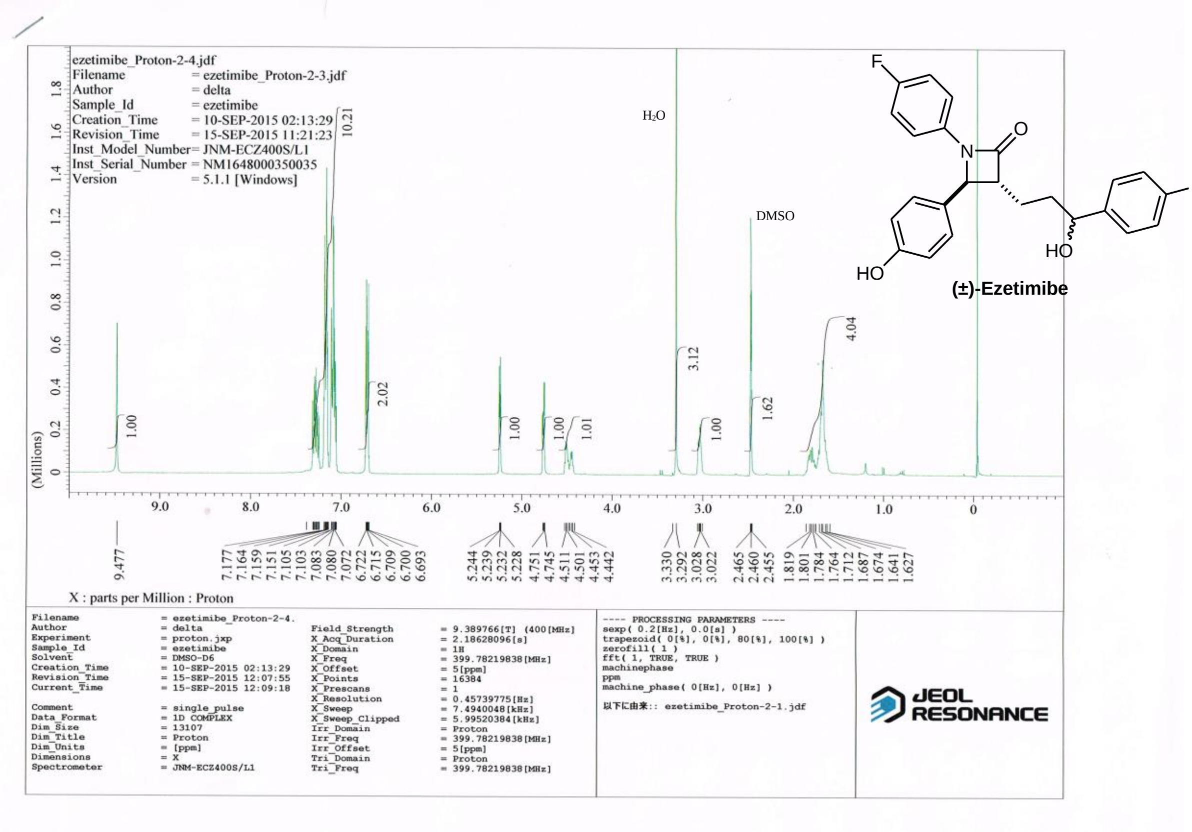

^{13}C NMR

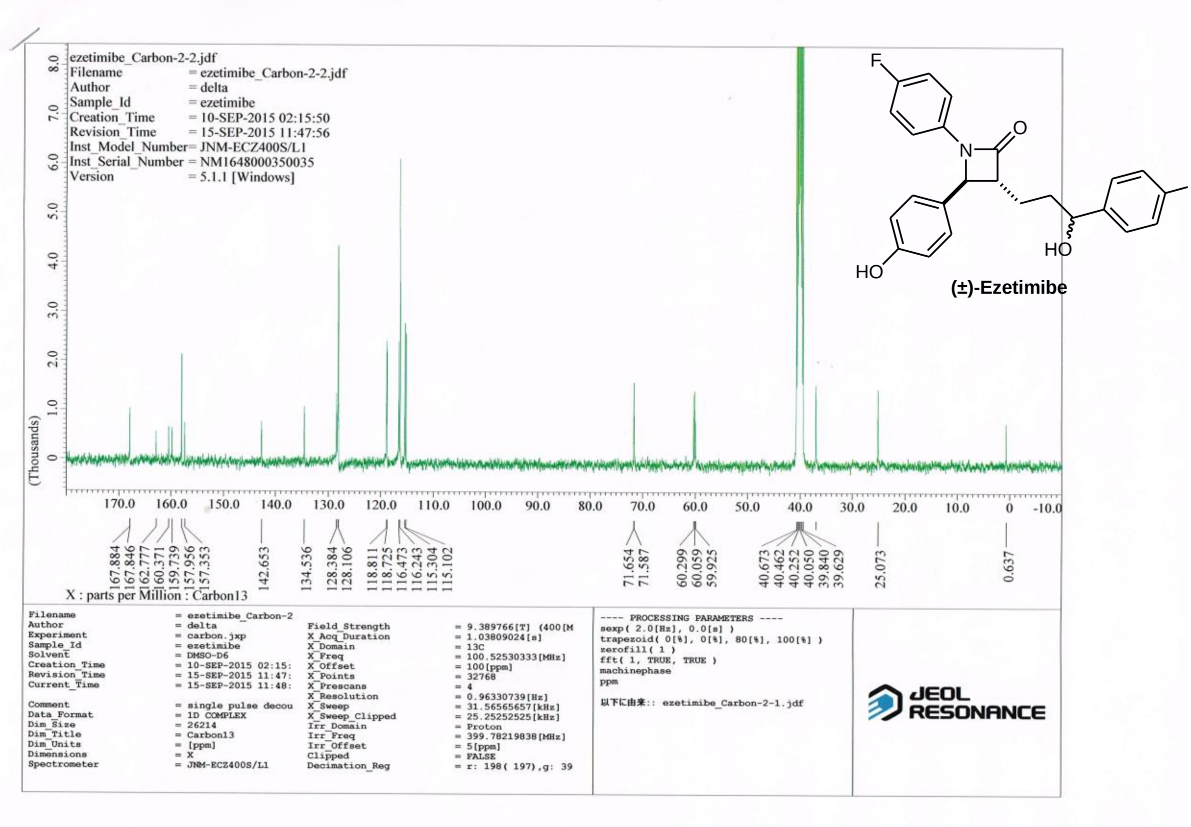

Supplement: File 1 — Experimental details, characterization of the compounds, X-ray crystallographic analysis of anti-3Ai, and 1H, 13C NMR spectra. [file Beilstein_J_Org_Chem-12-1608-s001.pdf]
